# Supplementary material for: The effects of genital myiasis on the diversity of the vaginal microbiota in female Bactrian camels
Source: BMC Vet Res. 2022 Mar 5;18:87. doi: 10.1186/s12917-022-03189-5 (PMC8897907; doi:10.1186/s12917-022-03189-5)
Supplement: Supplementary file 5 — Additional file 5. [file 12917_2022_3189_MOESM5_ESM.zip › MPL201709200_16s_yy/Treat1/css/js_css/jsPDF-master.zip!/jsPDF-master/docs/specs_utils_reference-server.js.html]

specs/utils/reference-server.js - Documentation


## Home

### Classes

- jsPDF

### Global

- addFont
- addMetadata
- addPage
- CapJoinStyles
- circle
- ellipse
- getFontList
- http
- lines
- lstext
- output
- rect
- roundedRect
- save
- setDisplayMode
- setDrawColor
- setFillColor
- setFont
- setFontSize
- setFontStyle
- setLineCap
- setLineJoin
- setLineWidth
- setPage
- setProperties
- setTextColor
- text
- triangle

# specs/utils/reference-server.js

```
'use strict'

/**
 * The reference server collects and saves reference PDFs for the tests.
 */
const http = require('http')
const PORT = 9090
const fs = require('fs')

// Create a server
const server = http.createServer((request, response) => {
  console.log(request.url)

  const wstream = fs.createWriteStream('./' + request.url)
  request.on('data', (chunk) => {
    console.log(chunk.length)
    wstream.write(chunk)
  })
  request.on('end', () => {
    wstream.end()
  })
  response.end('Test has sent reference PDF for ' + request.url)
})

// Lets start our server
server.listen(PORT, () => {
  console.log(`Server listening on: http://localhost:${PORT}`)
})
```

  

Documentation generated by JSDoc 3.4.2 on Sat Oct 08 2016 21:59:09 GMT+0100 (BST) using the docdash theme.
